# Transcriptomic profiling reveals key early response genes during GDF6‐mediated differentiation of human adipose‐derived stem cells to nucleus pulposus cells

**DOI:** 10.1002/jsp2.1315

**Published:** 2024-01-19

**Authors:** Hamish T. J. Gilbert, Francis E. J. Wignall, Leo Zeef, Judith A. Hoyland, Stephen M. Richardson

**Affiliations:** ^1^ Division of Cell Matrix Biology and Regenerative Medicine, School of Biological Sciences, Faculty of Biology, Medicine and Health University of Manchester, Manchester Academic Health Sciences Centre Manchester UK; ^2^ Bioinformatics Core Facility, Faculty of Biology, Medicine & Health University of Manchester Manchester UK

**Keywords:** adipose‐derived stem cell, differentiation, intervertebral disc, nucleus pulposus, regenerative medicine, transcriptomics

## Abstract

**Background:**

Stem cell‐based therapies show promise as a means of repairing the degenerate intervertebral disc, with growth factors often used alongside cells to help direct differentiation toward a nucleus pulposus (NP)‐like phenotype. We previously demonstrated adipose‐derived stem cell (ASC) differentiation with GDF6 as optimal for generating NP‐like cells through evaluating end‐stage differentiation parameters. Here we conducted a time‐resolved transcriptomic characterization of ASCs response to GDF6 stimulation to understand the early drivers of differentiation to NP‐like cells.

**Methods:**

Human ASCs were treated with recombinant human GDF6 for 2, 6, and 12 h. RNA sequencing and detailed bioinformatic analysis were used to assess differential gene expression, gene ontology (GO), and transcription factor involvement during early differentiation. Quantitative polymerase chain reaction (qPCR) was used to validate RNA sequencing findings and inhibitors used to interrogate Smad and Erk signaling pathways, as well as identify primary and secondary response genes.

**Results:**

The transcriptomic response of ASCs to GDF6 stimulation was time‐resolved and highly structured, with “cell differentiation” “developmental processes,” and “response to stimulus” identified as key biological process GO terms. The transcription factor ERG1 was identified as a key early response gene. Temporal cluster analysis of differentiation genes identified positive regulation NP cell differentiation, as well as inhibition of osteogenesis and adipogenesis. A role for Smad and Erk signaling in the regulation of GDF6‐induced early gene expression response was observed and both primary and secondary response genes were identified.

**Conclusions:**

This study identifies a multifactorial early gene response that contributes to lineage commitment, with the identification of a number of potentially useful early markers of differentiation of ASCs to NP cells. This detailed insight into the molecular processes in response to GDF6 stimulation of ASCs is important for the development of an efficient and efficacious cell‐based therapy for intervertebral disc degeneration‐associated back pain.

## Graphical abstract

This study identifies a multifactorial early gene response that contributes to lineage commitment, with the identification of a number of potentially useful early markers of differentiation of adipose‐derived stem cells to nucleus pulposus cells. This detailed insight into the molecular processes in response to GDF6 stimulation of ASCs is important for the development of an efficient and efficacious cell‐based therapy for intervertebral disc degeneration‐associated back pain.

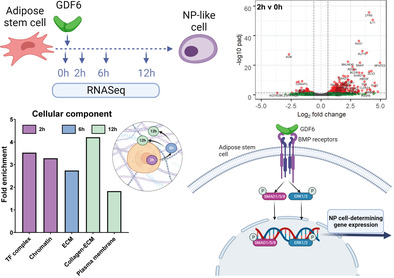

## 1 INTRODUCTION

Chronic back pain caused by intervertebral disc (IVD) degeneration remains one of the leading causes of long‐term morbidity and has a substantial socioeconomic impact globally.^1^ However, despite the clear imperative, there remains a lack of treatment options which offer long‐term restoration of functional IVD tissue. Substantial research has been directed at developing novel therapeutic strategies which could restore IVD tissue integrity and function, in particular restoration of healthy extracellular matrix (ECM) within the central nucleus pulposus (NP) region of the IVD. To date, a range of cell‐based regenerative medicine strategies have been proposed, with the application of multipotent adult stem cells comprising a substantial area of interest.^2^

In such stem cell‐based approaches, growth factors are often used to control differentiation and ultimately appropriate ECM synthesis. Most notably, members of the transforming growth factor beta (TGFβ) superfamily of growth factors, including TGFβ, growth differentiation factor 5 and 6 (GDF5/GDF6; also known as BMP14 and 13, respectively) have been used, to stimulate discogenic differentiation (i.e., differentiation to an NP‐like phenotype) of both bone marrow‐derived mesenchymal stem cells (MSCs) and adipose derived stem cells (ASCs).^3^, ^4^, ^5^

Our previous research suggests ASCs are superior in their ability to differentiate into NP‐like cells and to form a matrix more akin to that of the healthy NP.^3^, ^6^, ^7^ Crucially, this differentiation was driven most efficiently through stimulation with GDF6, rather than either GDF5 or TGFβ. We further demonstrated that this enhanced response in ASCs was due to significantly higher expression of the key GDF6 receptor sub‐unit BMPR2 in ASCs compared to MSCs.^7^ This increased BMPR2 expression was linked to increased SMAD1/5/8 and Erk1/2 activity in ASCs, which resulted in the enhanced cellular response to GDF6, primarily in terms of increases in expression of aggrecan and type 2 collagen genes. As such, ASCs stimulated with GDF6 may offer the potential to restore a healthy NP ECM in patients with IVD degeneration. However, while our previous investigations into the end‐stage of the GDF6‐induced differentiation response of ASCs has revealed an appropriate phenotype and we have identified key early signaling events, a more detailed understanding of the initial stages of cellular differentiation in response to GDF6 is important in developing an efficient and reproducible cellular therapy. Characterization of these early gene expression responses to GDF6 treatment is important, as it has the potential to identify novel factors which may aid in generation of the desired cellular phenotype or enable their use synergistically with GDF6 for an improved (higher efficiency/production of desirable proteins) cellular response.

Thus, our aim in this study was to assess the response of ASCs to GDF6 in detail, focusing on the early gene expression changes occurring in the first hours following stimulation. We report, using RNA sequencing and bioinformatics, the time resolved ASC response to GDF6. We further identify cellular processes and transcription factors activated by GDF6 and elucidate the signaling pathways involved in the response.

## 2 METHODOLOGY

### 2.1 Cell extraction and culture

All experiments and procedures carried out in this study were performed with relevant approval from the National Research Ethics Service and The University of Manchester. Adipose tissue was obtained from donors undergoing hip replacement surgery, with each donor providing full written informed consent. The ASC extraction process was undertaken as previously described.^7^ Donor ASCs used for experimentation (*n* = 9; Table S1) were expanded in culture medium containing: 110 mg/L sodium pyruvate, 1000 mg/L glucose, and further supplemented with final concentrations of 10% (v/v) FBS, 2 mM GlutaMAX (Life Technologies), 50 μg/mL ascorbate, 100 U/mL penicillin, 100 μg/mL streptomycin, and 0.25 μg/mL amphotericin (subsequently termed standard culture medium). ASCs were grown in ambient incubator conditions (37°C, 5% CO_2_, 20% O_2_) until sufficient cell numbers were obtained. All donors were used between passages 3–5 and around 14 days in culture.

### 2.2 GDF6 stimulation

ASCs were seeded in flasks or multiwell plates at a density of 7.5 × 10^3^ cells/cm^2^ (70%–80% confluence) and allowed to adhere for 24 h. Cells were washed twice in PBS and serum starved in FBS‐free standard culture medium (SFM) for a further 24 h. On the day of stimulation, medium was aspirated, cells washed twice in PBS and stimulated with 100 ng/mL of GDF6 (Peprotech) in SFM as previously described.^3^, ^6^, ^7^ Stimulation was performed for a variety of durations which are described for each experiment and forms the temporal‐nature of these studies.

To assess the importance of intracellular signaling mechanisms on the early response genes following GDF6 stimulation, Smad1/5/8 phosphorylation was inhibited by pre‐incubation with 10 μM dorsomorphin (Merck, cat no. 171261) and ERK1/2 phosphorylation was inhibited by pre‐incubation with 10 μM U0126 (Merck, cat no. 662009).^7^ Furthermore, protein translation was inhibited by pre‐incubation with 10 μg/mL cyclohexamide (Sigma, cat no. C4859) to help elucidate whether gene expression was directly downstream of GDF6‐mediated signaling or reliant upon de novo protein synthesis. In all experiments, following stimulation, cells were washed twice with ice‐cold PBS before either protein or RNA extraction.

### 2.3 RNAseq

Total RNA was extracted using TRIzol according to the manufacturers protocol, then resuspended in 20 μL TE buffer. Concentration, quality, and purity of RNA were determined using a Qubit (Invitrogen) fluorometer and Agilent 2200 Tapestation. QC‐checked total RNA for the three donors and four timepoints (0, 2, 6, and 12 h) were submitted to the Genomics Technologies Core Facility, University of Manchester, for RNAseq. Libraries were generated using the TruSeq® Stranded mRNA assay (Illumina, Inc.) according to the manufacturer's protocol. Briefly, total RNA (0.1–4μg) was used as input material from which polyadenylated mRNA was purified using poly‐T, oligo attached, magnetic beads. The mRNA was then fragmented using divalent cations under elevated temperature and then reverse transcribed using random primers into first strand cDNA. Second strand cDNA was then synthesized using DNA polymerase I and RNase H. Following a single “A” base addition, adapters were ligated to the cDNA fragments, and the products then purified and enriched by PCR to create the final cDNA library. Adapter indices were used to multiplex libraries, which were pooled prior to cluster generation using a cBot instrument. The loaded flow‐cell was then paired‐end sequenced (76 + 76 cycles, plus indices) on an Illumina HiSeq4000 instrument. Finally, the output data was demultiplexed (allowing one mismatch) and BCL‐to‐Fastq conversion performed using Illumina's bcl2fastq software, version 2.17.1.14. Unmapped paired‐end sequences from an Illumina HiSeq4000 sequencer were tested by FastQC. Sequence adapters were removed and reads were quality trimmed using Trimmomatic_0.36.^8^ The reads were mapped against the reference human genome (hg38) and counts per gene were calculated using annotation from GENCODE 31 using STAR_2.5.3 (PMID: 23104886).

### 2.4 Bioinformatic analysis of RNAseq data

The DESeq2 bioconductor package was used for processing the dataset and measuring differential gene expression.^9^ Negative binomial generalized linear models for each of the genes and Wald tests for differential expression analysis (i.e., timepoint comparisons using paired analysis) were used for significance testing, with *p*‐value adjusted for multiple testing (*p*_adj_ values) reported using Benjamini‐Hochberg multiple comparison corrections. Differential expression gene lists were determined using stimulatory timepoint (2, 6, 12 h) comparisons with unstimulated control (0 h) using *p*_adj_<0.05 and ≥1.5‐fold change cut‐offs. Specific gene searches were performed for important NP, osteogenic, and adipogenic genes.

Principle component analysis (PCA) was performed using DESeq2's “plotPCA” function to determine hierarchical variance of the top 500 genes based on highest row variance (i.e., across timepoints). The VSDEGMatrix function from the Vidger package was used to create the differential expression matrix for each available timepoint comparison. The Enhanced Volcano Package was used to create volcano plots for each timepoint compared to 0 h control and applying *p*_adj_<0.05 and ≥1.5‐fold change cut‐offs.

The MetaboAnalyst web tool was used to generate the gene expression heat map based on differentially expressed genes (*p*_adj_<0.05 and ≥1.5‐fold change) and the normalized counts for each timepoint compared to the 0 h control.

The gene ontology (GO) web tool g:Profiler was utilized for transcription factor analysis, while for identification of cellular components and biological processes, AmiGO2 was used. In both circumstances, differential gene lists were uploaded into the web tool. The three separate differential gene expression lists (2 h vs. 0 h, 6 h vs. 0 h, 12 h vs. 0 h), obtained from DESeq2 analysis, used *p*_adj_<0.05 and ≥1.5‐fold change cut‐offs. AmiGO2 was used to determine biological processes and cellular components, with the parental and top 10 child GO terms by fold enrichment belonging to cell differentiation and developmental process being identified for each timepoint.

Individual genes were grouped in clusters determined by K‐means clustering analysis that represent similar patterns of gene expressional changes over the experimental time course. These genes were then split into 12 clusters using the MaxdView software and Manhattan distance approach.

IPA was used to understand gene interactions, in a time dependent manner while also being able to evaluate upstream regulators of the dataset. The upstream regulators function was used to interrogate the transcription factor EGR1 identified from GO analysis and overlaying the 2 h versus 0 h differential gene expression values.

### 2.5 Real‐time PCR analysis

RNA was extracted using TRIzol according to the manufacturers protocol and resuspended in 20 μL TE buffer. Concentration and purity was determined using Nanodrop spectrophotometer and then the ABI high‐capacity cDNA reverse transcription kit and manufacturers protocol (ThermoFisher) was used to convert sample RNA to cDNA. Real‐time quantitative PCR (qPCR) was used to determine specific gene expression (Table S2) using either SYBR Fast Green (Applied Biosystems) or TaqMan™ Fast Advanced Master Mix (ThermoFisher). For each biological sample investigated, 2 μL of cDNA (5 ng/μL) was loaded in triplicate into reaction wells of 96‐well plate containing 8 μL of reaction mixture containing master mix, primers (plus probe for Taqman assays) and molecular grade water. Plates were run on a StepOnePlus real‐time PCR machine. Data were analyzed using 2 − ΔCt by calculating relative expression of target gene to the reference genes GAPDH and EIF2B1. qPCR was performed on both the samples used in the RNAseq experiments (cohort 1; *N* = 3) and a second cohort (cohort 2; *N* = 6). For statistical analysis (using GraphPad Prism) of cohort 1 and 2 datasets that both included a single independent variable (timepoint), repeated measures One‐way ANOVA Friedman test with Dunn's multiple comparison correction was applied. Repeated measures included matching of samples across the independent variable. Multiple comparisons were made between control (0 h) and experimental (stimulated timepoints) groups and determined as significant if *p*_adj_<0.05.

### 2.6 Western blotting

Protein extraction and Western blot analysis was carried out as previously described.^7^ Briefly, medium was aspirated and cells washed twice with ice cold PBS. Cells were lysed using RIPA lysis buffer with 1× protease and phosphatase inhibitor cocktail (ThermoFisher) and centrifuged 10 000 g at 4°C for 10 mins to remove cell debris. Sample protein concentration was determined using Pierce™ BCA Protein Assay Kit (ThermoFisher). Protein samples were frozen at −80°C until use.

For Western blot analysis, Life Technologies Bolt system and protocol was used throughout. For each sample, 20 μg of cell lysate was loaded into the wells of a 4%–12% Bis/Tris Bolt gel and separated by sodium dodecyl sulfate‐polyacrylamide gel electrophoresis (SDS‐PAGE). Proteins were transferred onto 0.45 μM PVDF membrane (ThermoFisher) and membranes blocked for 2 h in blocking buffer (5% BSA or 5% milk in TBS‐T) at room temperature on a rocking platform. Blocking buffer was removed and membranes incubated with primary antibody in blocking buffer overnight at 4°C on a rocking platform. The following antibodies were used: anti‐Smad1 (Cell Signalling 6944, 1:1000); anti‐pSmad1/5/8 (Cell Signalling 13 820, 1:1000); anti‐BMPR2 (ThermoFisher MA5‐15827, 1:1000); anti‐EGR1 (Abcam ab55160, 1:500); and anti‐GAPDH (Calbochem CB1001, 1:15000).

Membranes were washed 3 × 10 min with TBS‐T and then incubated with anti‐mouse (Cell Signalling 58 802, 1:15000) or anti‐rabbit (Cell Signalling 93 702, 1:15000) secondary antibodies in blocking buffer for 1 h at room temperature on rocking platform. Membranes were washed 3 × 10 min with TBS‐T. For protein detection, 2 mL of enhanced chemiluminescent reagent (PerkinElmer) was added to each membrane for 1 min, dabbed dry and sealed in polyethylene before being exposed to photographic film. Band densitometry was determined using image J software and band densities were further quantified relative to respective GAPDH bands to normalize samples to protein loading variances.

## 3 RESULTS

### 3.1 Confirmation of ASC response to GDF6 and RNASeq quality control

Expression of BMPR2 was confirmed in all three donor ASC samples (Figure S1), as was SMAD phosphorylation 1 h after GDF6 stimulation (Figure S1). A quality control check on total RNA from all three donors at all four RNAseq timepoints (0, 1, 6, and 12 h) prior to RNAseq submission revealed high RNA integrity (RIN_e_ 9.9 or greater) and high purity (Nanodrop ratios 1.8–2.2) (Figure S2). A quality control check of the raw read data (FASTQ) generated from RNAseq confirmed that for all samples the uniquely mapped reads was ≥95%, reads mapped to multiple loci was <4%, reads unmapped due to mismatches was 0% and the percentage of reads counted into genes was ≥90% (Tables S3–S5).

### 3.2 Time resolved transcriptomics of ASCs treated with GDF6 reveals changes in expression of genes associated with differentiation and secretion

Principal component analysis (PCA) revealed that samples were clustered by donor in PC1 and 2, with variation between timepoints apparent in PC3 and 4, and post‐stimulation timepoint separation from 0 h identifiable in PC5 and 6 (Figure S3).

**FIGURE 1:**
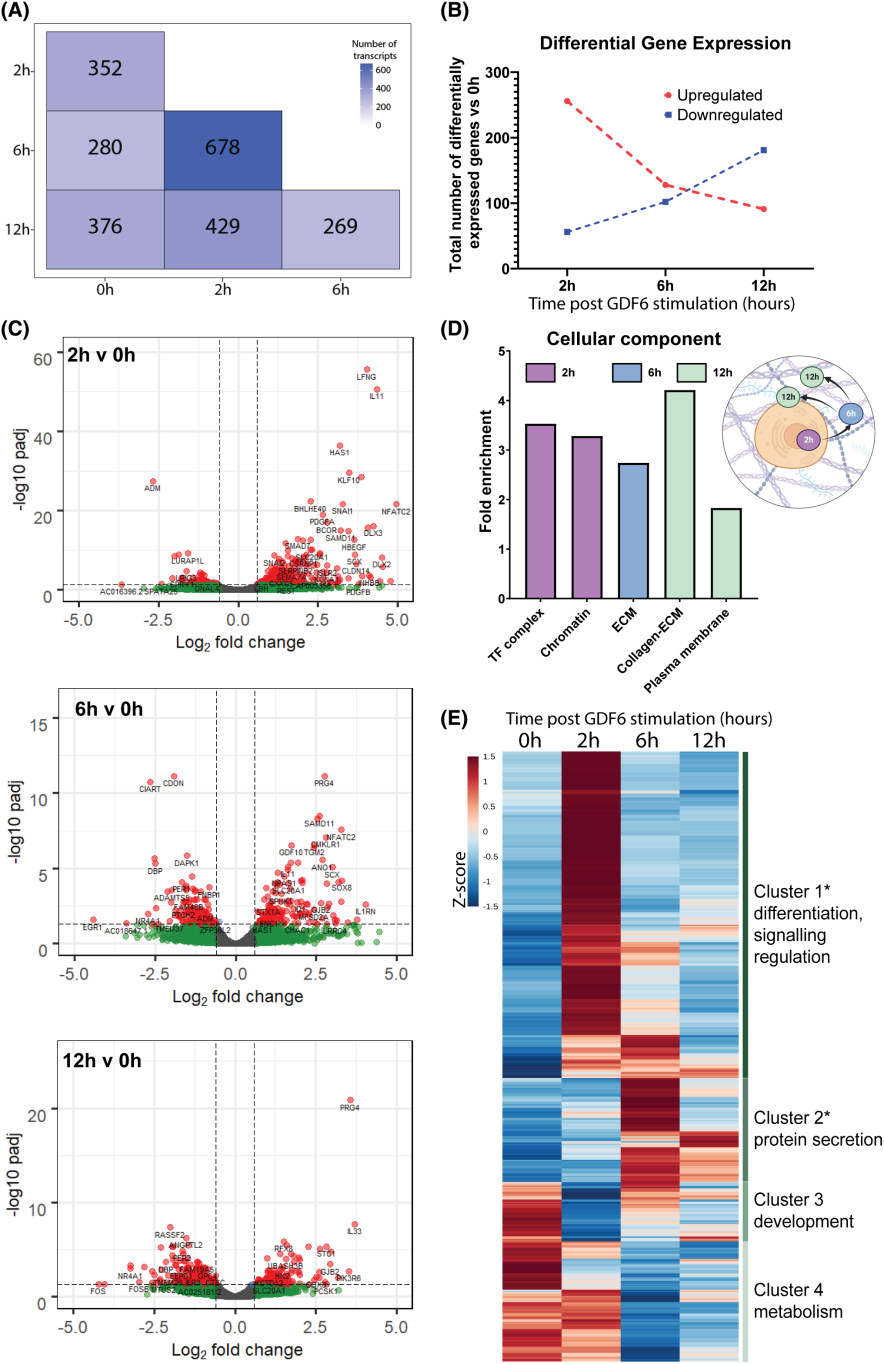
Bioinformatic analysis of RNAseq dataset. (A) Number of differentially regulated transcripts identified in RNA sequencing of ASCs treated with GDF6 for 2, 6, and 12 h and normalized to untreated 0 h control. (B) Total number of genes up (red) or down (blue) regulated at each timepoint post GDF6 stimulation, relative to 0 h. (C) Volcano plots display differentially expressed genes at each timepoint normalized with 0 h control. *N* = 3, *p*_adj_ <0.05, fold change >1.5, paired‐analysis Wald test with Benjamini‐Hochberg multiple comparison corrections. (D) Gene ontology analysis for “Cellular Components” of RNA sequencing data and their respective fold enrichment scores. Schematic of cellular locations of gene set for each timepoint as determined by cellular component analysis. (E) Heat‐map of hierarchical cluster analysis of RNA sequencing data clustered by timepoint on the X axis and by Z‐score on the Y axis. The four main gene clusters were analyzed using AmiGO2 and labeled based on the prevalent significant GO terms associated. High Z score (red) and low Z‐score (blue), with mean *Z*‐scores of the three donor scores displayed.

Comparison of differential gene expression analysis at each timepoint following GDF6 stimulation compared to unstimulated control (0 h) revealed similar numbers of regulated genes (2 h vs. 0 h =352, 6 h vs. 0 h = 280, 12 h vs. 0 h = 376) (Figure 1A). However, the ratio between up and down regulated genes differed between timepoints, with genes at 2 h versus 0 h upregulated (5:1), genes at 6 h versus 0 h having an even split (1:1) and genes at 12 h versus 0 h being downregulated (1:2) (Figure 1B). Genes with the greatest fold changes and significance at 2 h versus 0 h (Figure 1C) included LFNG (known for regulating cell fate decisions during development), genes associated with cell differentiation (SNAI1, SNAI2, SCX), IL11 (also known as adipogenesis inhibitory factor), KLF10 (also known as TGFβ‐inducible early growth response protein) and HAS1 (hyaluronic acid synthase). At 6 h versus 0 h genes associated with matrix remodeling (PRG4, GDF10, CDON, ADAMTS5) were regulated. PRG4, which has previously been identified in the IVD,^10^, ^11^ was found to be the most significantly upregulated gene at 6 h versus 0 h and 12 h versus 0 h.

GO analysis assessment for “cellular component” revealed a temporal shift from intracellular to extracellular genes (Figure 1D). Analysis of 2 h versus 0 h showed enrichment for “transcription factor complex” and “chromatin,” while the 6 h and 12 h timepoints were both enriched for ECM‐related cellular component terms and 12 h also being enriched for plasma membrane terms.

Hierarchical clustering (Figure 1E) revealed four main clusters of genes, with clusters 1 & 2 being significant (*p* < 0.05). Cluster 1 represented genes with upregulation at 2 h and related to differentiation and signaling regulation (AmiGO2 analysis). Cluster 2, characterized by gene upregulation at 6 h, was associated with protein secretion. Cluster 3 was associated with upregulation of development genes at 0 h and downregulation at the other timepoints, while cluster 4 was related to downregulation of metabolism genes over time.

### 3.3 Regulation of EGR1 transcription factor in ASCs response to GDF6

**FIGURE 2:**
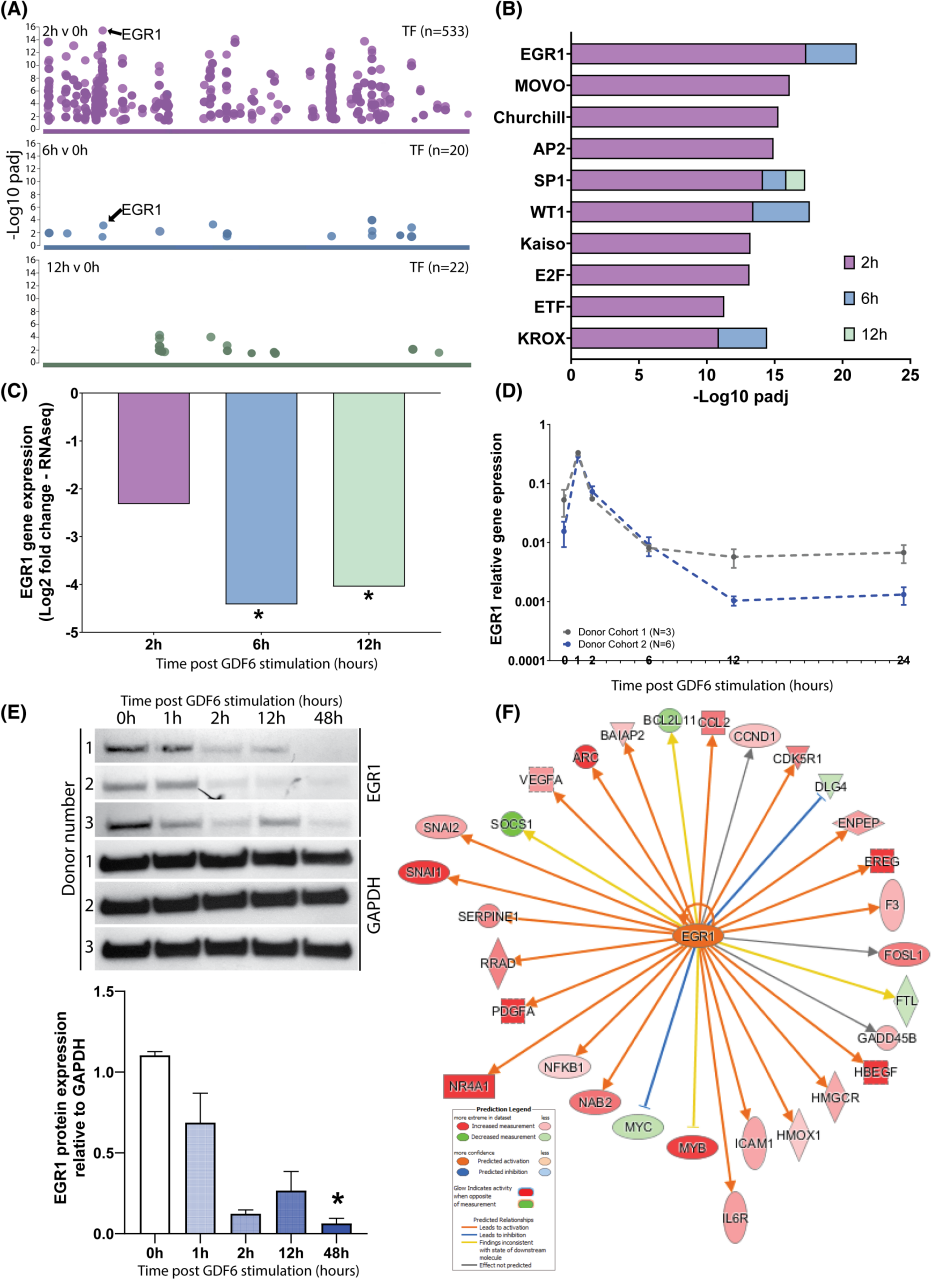
Transcription factor gene ontology and expression analysis. (A) Transcription factor (TF) analysis of RNA sequencing dataset. Scatter plots show the number of TFs for each timepoint and their corresponding –log10 *p*_adj_ value. (B) The top 10 most enriched transcription factors (−log10 *p*_adj_ value) at 2 h versus 0 h (purple), 6 h versus 0 h (blue), and 12 h versus 0 h (green), with the transcription factor of interest (EGR1) highlighted. *N* = 3, *p*_adj_<0.05, Bonferroni correction for multiple testing. (C) RNAseq analysis of temporal EGR1 expression at 2, 6, and 12 h post GDF6 stimulation. (D) qPCR validation of EGR1 in the RNAseq cohort (cohort 1, gray) and the additional cohort (cohort 2, blue). Expression values are relative to that of the mean of the two reference genes GAPDH and EIF2β1. *N* = 3 (cohort 1), *N* = 6 (cohort 2), *p*_adj_<0.05, repeated measures One‐way ANOVA Friedman test with Dunn's multiple comparison correction. (E) Western blot analysis of EGR1 relative to GAPDH in three different donors stimulated with GDF6 over time. Bar graph displays densitometry quantification of blot, with a significant decrease in EGR1 expression at 48 h. *N* = 3, *p* < 0.05, fold change >1.5, paired‐analysis Wald test with Benjamini‐Hochberg multiple comparison corrections. (F) Ingenuity pathway analysis of RNA sequencing data at 2 h post GDF6 treatment. Upstream regulatory assessment of EGR1. The predicted activation and known molecule interactions are shown. Genes known to play roles in stem cell differentiation are expanded.

GO analysis was used to identify transcription factors using known regulatory binding motifs to the genes within the dataset (Figure 2A). The 2 h versus 0 h dataset was associated with a substantially higher number of transcription factors (*n* = 533) compared with the 6 h versus 0 h (*n* = 20) and 12 h versus 0 h (*n* = 22) datasets. EGR1 was the most significant transcription factor at 2 h versus 0 h and 6 h versus 0 h (Figure 2B). EGR1 was highlighted due to its documented roles in stem cell response and differentiation.^12^, ^13^ Other TFs included SP1 (cell differentiation), Churchill (mediates FGF signaling), AP2 (proliferation and the suppression of terminal differentiation during embryonic development), WT1 (tumor suppressor gene), Kaiso (regulator of P53), E2F (cell cycle), ETF (EGFR stim), KROX (also known as EGR2) (Figure 2B).

The expression levels of EGR1 were assessed using the RNAseq dataset (Figure 2C) and the temporal expression changes in EGR1 validated both in the same cohort (donor cohort 1, *N* = 3) and a second larger cohort (donor cohort 2, *N* = 6) using qPCR (Figure 2D). EGR1 was significantly downregulated at 6 h and 12 h in the RNAseq analysis (Figure 2C), with a similar trend being seen in both cohorts in qPCR analysis. EGR1 protein levels also decreased over time, significantly so by 48 h (Figure 2E). Pathway analysis (Figure 2F) predicted that EGR1 was activated and that it activated genes also known to play roles in differentiation, including SNAI1 and SNAI2 (embryonic development), NR4A1 (inhibitor of PPARG), and IL6R (inflammation).

### 3.4 GO analysis identifies “cell differentiation,” “developmental processes,” and “response to stimulus” terms

**FIGURE 3:**
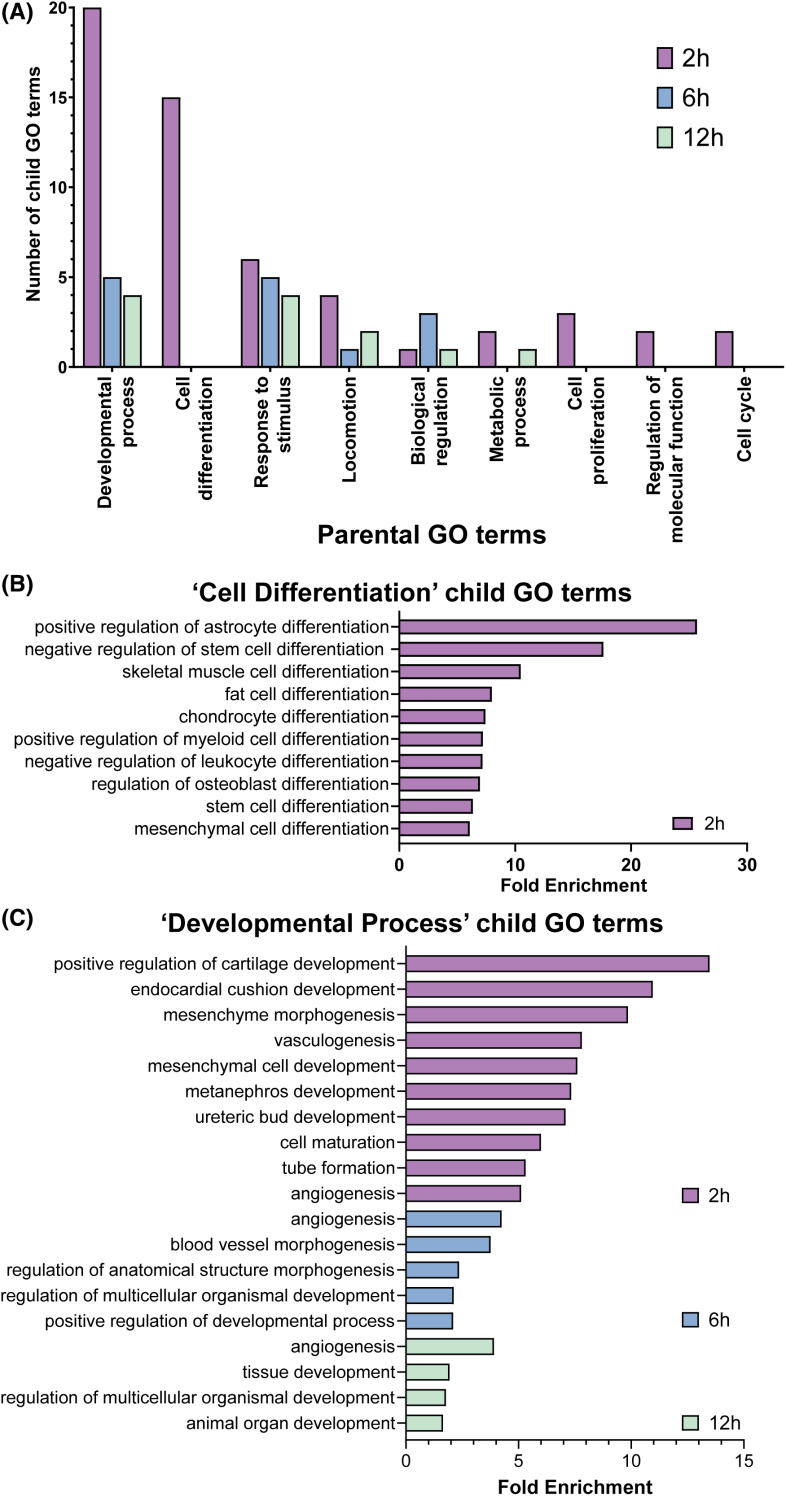
Gene ontology (GO) analysis of biological processes. (A) Bar chart showing parental GO terms and the number of child GO terms belonging to each parental GO term for each timepoint post GDF6 stimulation. (B) Bar chart showing the top 10 child GO terms for each timepoint in regards to fold enrichment for “cell differentiation” related terms. (C) Bar chart showing the top 10 child GO terms for each timepoint in regards to fold enrichment for “developmental process” related terms.

GO analysis was employed to identify the biological processes enriched at each timepoint (Figure 3). The child GO terms identified for each timepoint were categorized into their respective parental GO terms, with the majority related to cell differentiation, developmental processes, and response to stimulus (Figure 3A). Over the timecourse, the number of biological processes decreased for all but “Response to stimulus” GO terms. The top 10 child GO terms ranked by fold enrichment for “cell differentiation” (Figure 3B) and “developmental process” (Figure 3C) were listed. Importantly, the three common lineages of MSC differentiation (“chondrocyte differentiation,” “fat cell differentiation,” and “regulation of osteoblast differentiation”) were among the top 10 cell differentiation terms. In addition, “positive regulation of cartilage development” was the highest developmental process term ranked by fold enrichment.

### 3.5 Characterization of lineage specification

**FIGURE 4:**
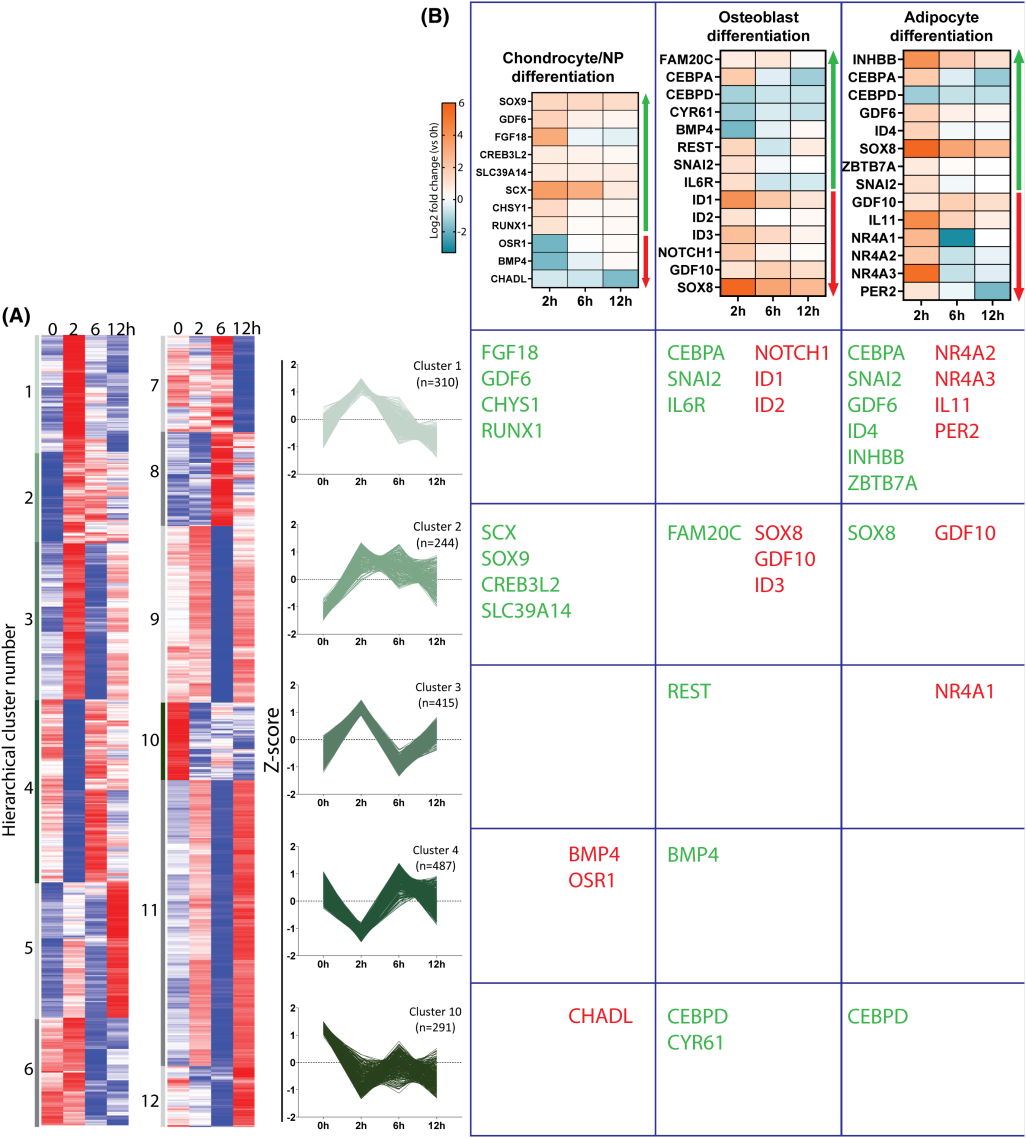
Differential gene expression analysis for genes belonging to chondrocyte/NP differentiation, osteoblast differentiation, and adipocyte differentiation GO terms. (A) K‐means clustering identified 12 independent cluster groups from the RNAseq dataset. Clusters are based on gene expression levels for independent timepoints (0, 2, 6, and 12 h) and grouped on similarity of gene expression changes over the 4 timepoints. Red represents high and blue represents low *Z*‐scores. *N* = 3, *p* < 0.05, fold change >1.5, paired‐analysis Wald test with Benjamini‐Hochberg multiple comparison corrections. (B) Table containing heat maps of differential gene expression at each timepoint for genes associated with chondrocyte/NP differentiation, osteoblast differentiation and adipocyte differentiation. Heat maps coloring depict log2‐fold change at each timepoint compared to 0 h with blue representing downregulation and orange representing upregulation of genes. The genes in each heat map are clustered based on whether they promote (green arrow) or inhibit (red arrow) differentiation. Genes from the three different gene ontology terms are assigned to the individual *K*‐means cluster that they belong. *N* = 3, *p*_adj_<0.05, fold change >1.5, paired‐analysis Wald test with Benjamini‐Hochberg multiple comparison corrections.

Using a combination of k‐means cluster analysis and differential expression heatmaps, early response genes associated with the GO terms of interest (chondrocyte/NP differentiation, osteoblast differentiation, and adipocyte differentiation) were investigated to give further insight into mechanisms of lineage specification (Figure 4). Most notably, genes that promote chondrocyte/NP differentiation were seen to be upregulated, with SOX9 and SCX being maintained over the entire time course (cluster 2). Chondrocyte/NP differentiation inhibitory genes (BMP4, OSR1, and CHADL) were downregulated, with CHADL being downregulated over the entire time course (cluster 10). Osteoblast differentiation generally showed upregulation of inhibitor and downregulation of promoter genes. ID1‐3 (clusters 1 and 2), genes known for inhibiting osteogenesis, were all upregulated while promoters of osteogenesis (BMP4 and CYR6; cluster 4 and 10, respectively) were downregulated over multiple timepoints. Adipocyte differentiation saw a combination of promotion and inhibition at 2 h, but most notably downregulation of CEBPA and CEBPD (two crucial transcription factors in adipogenesis) by 6 h and 12 h. Furthermore, all three NR4A transcription factors (inhibitors of adipogenesis), were upregulated at 2 h.

Several genes are known to play roles in multiple differentiation lineages. GDF10, for example, inhibits both osteogenesis and adipogenesis^14^, ^15^ while having positive roles in chondrogenic differentiation and remodeling of ECM.^16^ Interestingly, GDF10 was upregulated over the entire time course (cluster 2). SOX8 (cluster 2), which was upregulated across the entire time course, is another example of multi‐lineage participation and is a positive regulator of chondrogenesis.^17^

K‐means cluster 2 describes genes that are upregulated and maintained over the 12 h time course. There were four genes that promote chondrocyte/NP differentiation in this cluster, while osteoblast differentiation and adipocyte differentiation had only one promoter each and one and three inhibitors, respectively. Cluster 10 describes genes where downregulation was maintained over the time course, which included genes that promote osteoblast and adipocyte differentiation. Most genes across chondrocyte/NP, osteoblast and adipocyte differentiation belonged to cluster 1 which describes the fast‐responding genes that were upregulated at 2 h and subsequently downregulated back to basal levels.

**FIGURE 5:**
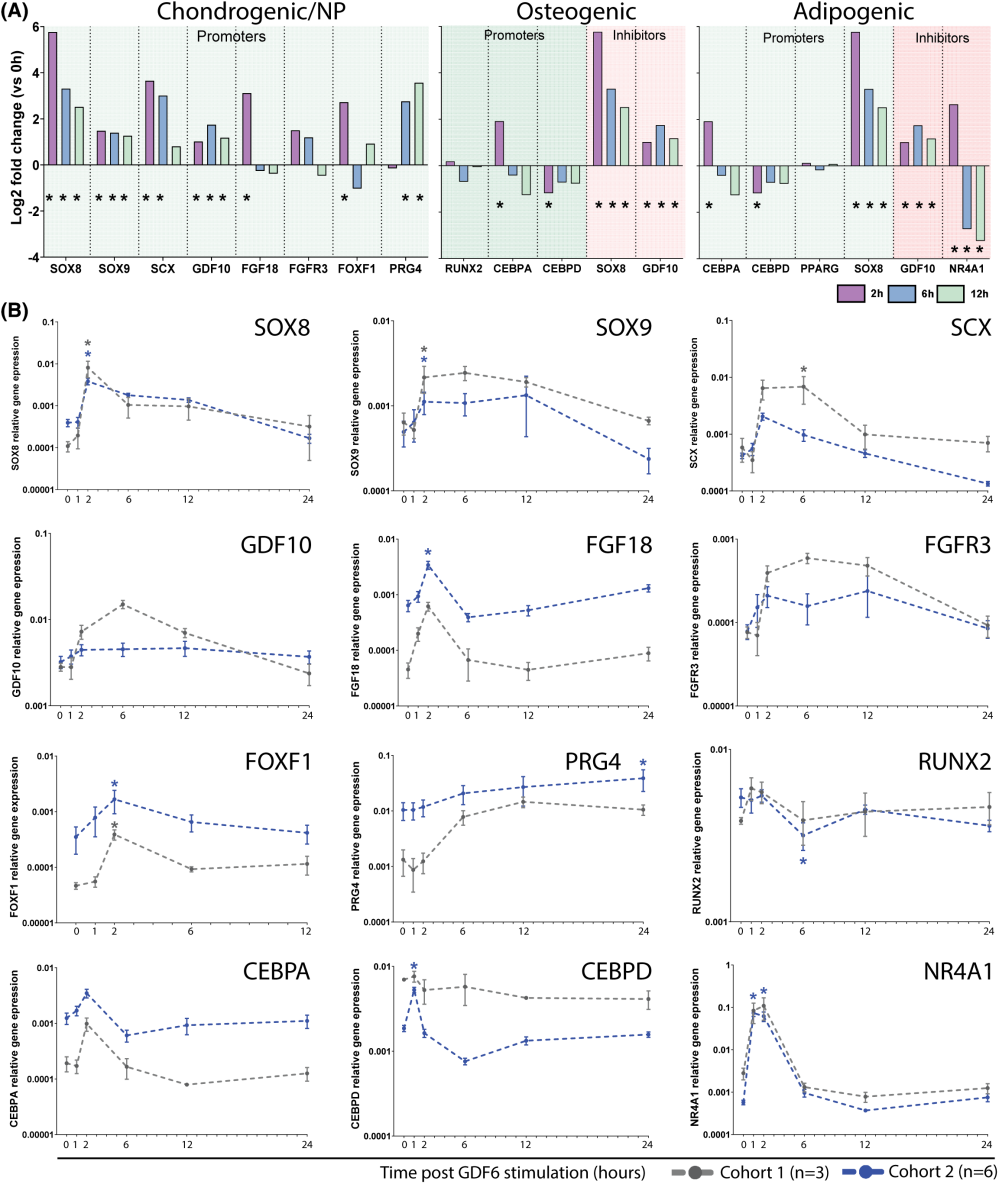
RNAseq and RT‐qPCR analysis of temporal trilineage‐related early response gene expression. (A) Bar chart shows RNAseq Log2‐fold change of mean normalized reads per gene at 2, 6, and 12 h compared with the mean of 0 h. Early response genes that influence chondrogenic/NP, adipogenic and osteogenic differentiation are indicated. Genes are split and highlighted if known for promoting (green) or inhibiting (red) differentiation. *N* = 3, *p*_adj_<0.05*, paired *T*‐test. RNAseq data bar charts represent fold change of mean normalized reads per gene for each timepoint compared to 0 h. (B) qPCR validation of early response genes in the original RNAseq cohort (cohort 1, *N* = 3, gray) and an additional cohort (cohort 2, *N* = 6, blue). Values are relative to the mean expression values of the reference genes GAPDH and EIF2β1. *p < 0.05 (color coded to cohort) compared with 0 h control, repeated measures One‐way ANOVA Friedman test with Dunn's multiple comparison correction.

Temporal changes in expression of promoter and inhibitor genes of chondrogenic/NP, osteogenic and adipogenic differentiation identified using RNASeq (Figure 5A) were further confirmed in both cell cohorts using qPCR (Figure 5B), with expression patters from qPCR analysis marching that seen in the RNAseq analysis.

### 3.6 Molecular signaling pathways involved in the early gene response to GDF6

**FIGURE 6:**
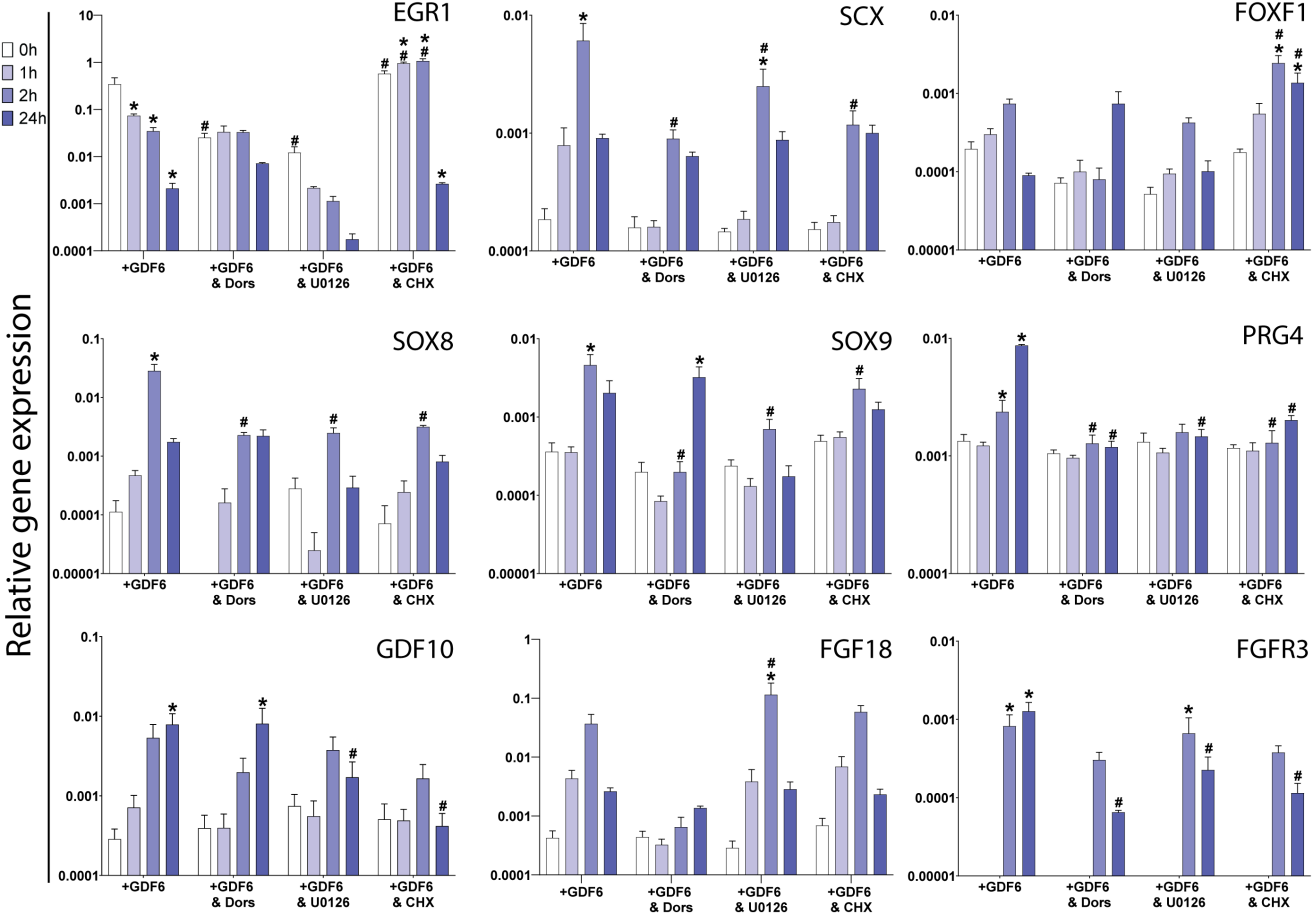
qPCR analysis of temporal early response gene expression in adipose stem cells (ASCs) stimulated with growth differentiation factor 6 (GDF6) while inhibited for Smad1/5/8, ERK1/2 and protein translation. Cohort 1 ASCs were treated with standard, serum‐free, stimulatory conditions (+GDF6, 100 ng/mL for 1 h, 2 h and 24 h) and inhibited with either dorsomorphin (Dors, Smad1/5/8 inhibitor), U0126 (ERK1/2 inhibitor) or cyclohexamide (CHX, protein translation inhibitor). 0 h represents the non‐GDF6 stimulated timepoint control for each group. Gene expression was quantified relative to GAPDH and EIF2b1. *N* = 3, *p < 0.05 for comparison with 0 h control in the same condition, #*p* < 0.05 for comparison with +GDF6 control at that same timepoint, repeated measures two‐way ANOVA and Dunnett's multiple comparison correction.

SMAD1/5/8, Erk1/2 and protein translation were inhibited prior to GDF6 stimulation to understand the roles of signaling pathways and de novo protein synthesis in regulation of identified early response genes (Figure 6).

The temporal downregulation of EGR1 identified following GDF6 stimulation was also observed with addition of the Erk1/2 inhibitor U0126, which was not observed following addition of the SMAD1/5/8 inhibitor Dorsomorphin. ERG1 was significantly upregulated at 1 h and 2 h with addition of the protein translation inhibitor cycloheximide, but then downregulated by 24 h.

SOX8, SOX9, SCX, FGFR3, and PRG4 expression were significantly reduced at their respective peak expression timepoint in all three inhibitory conditions compared to the GDF6 stimulation only control. FOXF1 temporal expression pattern was affected by inhibition or SMAD1/5/8 and protein translation, but not Erk1/2 inhibition. FGF18 expression was not significantly altered by inhibition of protein translation but did show a significant increase in expression with Erk1/2 inhibition at 2 h compared to 0 h, while SMAD1/5/8 inhibition demonstrated minimal temporal expression change. GDF10 expression was significantly reduced in the Erk1/2 and protein translation groups at 24 h, while SMAD1/5/8 inhibition showed a similar significant 24 h increase in GDF10 expression to the GDF6 stimulation only control.

## 4 DISCUSSION

We have previously demonstrated that human ASCs, when stimulated with GDF6, differentiate into a cell type akin to that of NP cells.^3^, ^6^, ^7^ We found 100 ng/mL GDF6 to be the optimal concentration (compared to 10 ng/mL or 1000 ng/mL) for driving in vitro differentiation and an equivalent concentration has been used by others in vitro and in vivo.^18^, ^19^ Furthermore, we have shown that high BMPR2 expression and GDF6‐initiated pSmad1/5/8 and pERK1/2 responses are paramount to differentiation efficiency.^7^ A number of NP‐specific markers are used to determine efficiency of end‐stage differentiation; however, there is a lack of understanding surrounding the early stages of differentiation and the more immediate gene responses to GDF6. Here we sought to gain a more detailed understanding of the key gene responses occurring in the first hours following GDF6 stimulation.

RNA sequencing and bioinformatics analysis of the early response of human ASCs to GDF6 stimulation identified a structured and time‐resolved cellular response. Understanding the cellular response of ASCs to GDF6 in a time‐resolved manner is important to fully understand the cellular mechanisms involved in lineage commitment. The number of upregulated genes in response to GDF6 was at its maximum at 2 h post‐stimulation, reducing to an equilibrium state at 6 h, and then the majority of genes were downregulated by 12 h. This temporal response is commonly seen with growth factor stimulations, likely due to the half‐life of growth factor/signaling molecules^20^ and negative feedback mechanisms.^21^ Smad7, an inhibitor of BMP signaling was upregulated at 2 h, providing an example where negative feedback could resolve the growth factor associated cellular response.^22^ Gene ontology analysis reporting “cellular component” identified involvement of the nucleus (transcription factor complex and chromatin) at 2 h, the ECM at 6 h and the cell‐ECM (collagen‐ECM and plasma membrane) at 12 h (Figure 1D), demonstrating a cellular response initiating from the nucleus and moving outwards. This time‐resolved response was also evident when using hierarchical clustering, with “differentiation, signalling regulation” activated at 2 h and “protein secretion” occurring at 6 h. The response at 2 h was enriched for a high number of TFs, which then reduced by 6 and 12 h. It is likely that the genes with increased expression at 2 h were primary response genes, and those genes increased at 6 and 12 h, secondary response genes. This prediction is supported by the enrichment of the genes increasing at 2 h with “nucleus” cellular component terms, and those genes increasing at 6 and 12 h with “extracellular region” cellular component terms, an enrichment pattern described previously for primary and secondary response genes, respectively.^23^, ^24^, ^25^ The high level of nucleus and TF activity at 2 h would represent initiation of the differentiation transcriptional cascade by GDF6 stimulus, while the 6 h/12 h timepoints reflected TF stabilization and progression of differentiation as suggested by the “collagen‐containing ECM” enrichment term at 12 h. This pattern of cellular response to growth factors has been reported previously, with “phases of differentiation” reported in MSCs committing to osteogenic and adipogenic lineages.^26^

Studying transcription factor enrichment provides insight into potentially important primary response genes related to growth factor stimulation. EGR1, a multifaceted, immediate early response gene, was found to be the most significantly enriched transcription factor in the RNAseq dataset at 2 h and 6 h. EGR1 plays roles in regulating components of matrix formation, is involved in stem cell quiescence and division, and is activated by multiple intracellular signaling pathways including ERK1/2 MAPK and Smad1/5/8 signaling.^13^, ^27^ Furthermore, EGR1 has been shown to mediate anabolic and catabolic responses to growth factors and inflammatory cytokines in NP cells,^28^ highlighting a potential role in relation to NP cell differentiation. EGR1 was predicted by IPA to be activated and was shown to regulate genes from the 2 h dataset that are involved in differentiation and matrix remodeling. Interestingly, qPCR analysis demonstrated that EGR1 was upregulated at 1 h, then downregulated at 6 h and 12 h. Due to EGR1s predicted activation, this suggests the peak of EGR1 gene expression within the first 2 h of differentiation, and then beyond 2 h is subject to down regulation via feedback loops. Overall, these findings suggest a critical role for EGR1 in the very early stages of ASC lineage commitment following GDF6 stimulation.

GDF6 is known to promote both chondrogenic and NP cell differentiation, and this was further supported by the term “chondrocyte differentiation” being the fifth highest “cell differentiation” GO term in regard to fold enrichment at 2 h. Furthermore, “positive regulation of cartilage development” was the highest developmental process GO term and “collagen‐containing extracellular matrix” cellular components was enriched at 12 h. Analyzing the genes associated with these GO terms revealed mechanisms of chondrogenic/NP promotion. This included upregulation of “chondrocyte differentiation” promoters (e.g., SOX9, GDF6, FGF18, and SCX) and downregulation of inhibitors (e.g., BMP4, CHADL). Sustained upregulation of SOX9 over the entire time course supports an important role for this transcription factor in driving NP cell differentiation, as has been shown previously.^29^, ^30^ SCX was significantly upregulated across the time course and is known to work synergistically with SOX9 during chondrogenesis.^31^ Combinatorial SOX9 and SCX expression might therefore be an essential early stage of NP differentiation.

FGF18 was significantly upregulated at 2 h and has been used previously as a supplementary factor for chondrogenic differentiation of MSCs^32^, ^33^ as well as an anabolic factor for NP cells.^34^ Interestingly, IPA analysis revealed interaction of FGF18 with FGFR3, which was also upregulated at 2 h and 6 h. FGF18 has been shown to signal through FGFR3 to promote chondrogenesis in MSCs^35^ and anabolism in chondrocytes,^36^ alluding to a potential mechanism of ASC differentiation toward NP cells. The “collagen‐containing extracellular matrix” GO term identified at 12 h revealed genes that are also associated with chondrogenic/NP differentiation. This included promoters (PRG4, GDF10, TIMP3, COL13A1, ACAN, TGFβ1, and ADAMTS4) that were largely upregulated and inhibitors (CHADL, ADAMTS5) that were downregulated across the time course, adding further credence to promotion of differentiation toward an NP‐like phenotype. PRG4, a marker of chondroprogenitor cells,^37^, ^38^ was the most significant gene across the whole dataset at 6 h and 12 h. Interestingly, PRG4 has been reported to be expressed 9‐fold higher in the NP compared with articular cartilage^39^ and has since been classified as a secondary NP marker.^30^ As such, significant upregulation of PRG4 as early as 6 h and its presence in the human NP could indicate an important molecule in the early stages of ASC differentiation toward an NP phenotype, demanding further investigation in longer‐term differentiation protocols.

During stem cell differentiation there is a balance of transcriptional activity that guides differentiation toward the correct lineage. Factors that promote differentiation toward one lineage may inhibit another.^40^ For example, SOX9, a promoter and master regulator of chondrogenesis, is also understood to inhibit promoters of adipogenesis (e.g., CEBPD) and osteogenesis (e.g., RUNX2).^41^, ^42^ This phenomenon explains why growth factors such as GDF6 have been shown to promote NP differentiation but also to inhibit osteogenic differentiation in MSCs.^43^ It was therefore interesting to find predominantly downregulation of promoters of osteogenic and adipogenesis differentiation and upregulation of their inhibitors.

CEBPA and CEBPD, which are crucial for early adipogenic lineage commitment (as well as playing a role in osteogenesis) were downregulated across the timecourse, while PPARG (the main driver of adipogenesis)^41^, ^44^ was not differentially expressed. SOX8 was found to be significantly upregulated across the entire time course and is known to be involved in promoting adipogenesis^45^ and inhibiting osteogenesis.^46^ GDF10 was also found to be upregulated across the entire time course. Importantly, GDF10 is also known to inhibit both adipogenesis and osteogenesis by suppressing PPARG^14^ and RUNX2^15^ expression, respectively; yet also promoting chondrogenesis.^16^ Therefore, with maintained temporal upregulation and involvement in multiple lineages, SOX8 and GDF10 might be playing important roles in mediating correct early lineage commitment. Future studies are needed to demonstrate the importance of cross‐lineage genes by understanding the impact on differentiation.

We have previously demonstrated that GDF6 stimulation of ASC differentiation to NP cells requires signaling primarily through Smad1/5/8, as well as Erk1/2. Therefore, specific signaling pathways were inhibited, including Smad1/5/8 (dorsomorphin) and ERK1/2 (U0126) signaling in ASCs stimulated with GDF6. Blocking of Smad1/5/8 and Erk1/2 pathways revealed that EGR1, SOX‐9, FOXF1, and FGF18 are directly regulated via Smad1/5/8 signaling, but not by Erk1/2. However, both SOX‐9 and FOXF1 showed a delayed response, rather than consistent inhibition, suggesting the potential for alternative regulatory pathways in the absence of Smad1/5/8. A number of other genes also had significantly reduced peak expression with both dorsomorphin and U0126, including: SOX8, SCX, FGFR3, and PRG4; suggesting these genes may be activated to some extent by both Smad1/5/8 and EKR1/2 signaling. Interestingly, all of these genes did show temporal expression changes, albeit reduced, under each inhibitory condition. This would suggest these genes are still being stimulated via the compensatory signaling mechanism (i.e., the one not being inhibited), resulting in the reduced but not nullified expression changes that were observed.

Protein translation was also inhibited (cyclohexamide) to ascertain whether specific gene responses were reliant upon the translation of other genes, thereby enabling a distinction to be made between the primary responses which do not require de novo protein synthesis for transcription regulation^24^ and secondary responses which do. Primary response genes that had common expression profiles with the non‐inhibited control included EGR1, FGF18, and FOXF1. The secondary response genes were characterized by demonstrating significantly decreased expression profiles when under cyclohexamide, which included: SOX8, SOX9, SCX, FGFR3, GDF10, and PRG4; and are therefore reliant upon the translation of primary response genes. SOX9, a commonly used NP marker alongside FOXF1,^30^ has been shown to be downregulated in embryonic mesenchyme development of FOXF1 homozygous knockout mice.^47^ FOXF1 could therefore be important in promoting SOX9 expression and the early stages of NP cell differentiation. FGF18 is also known to promote SOX9 expression in chondrogenesis through ERK signaling^48^ as well as FGFR3 expression.^49^ Our data showed increased FGF18 gene expression by 4 h, suggesting FGF18 could therefore be involved in sustaining SOX9 expression at the later timepoints through FGFR3. As well as being promoted directly from primary response genes, secondary response genes can also be regulated by other secondary response genes, as may be the case with SCX promoting SOX9 expression,^31^ while both GDF10 and SOX8 have been shown to be SOX9‐dependent in chondrocytes^50^ and during MSC chondrogenesis,^17^ respectively. The secondary response genes showed some temporal expression changes under cyclohexamide treatment which could be attributed to inefficiency of the inhibitor. Nonetheless, expression was significantly lower at peak timepoints in each case which strongly suggests their expression is translation‐dependent and are therefore likely to act as secondary response genes in GDF6‐stimulated ASCs.

## 5 CONCLUSIONS

This study has characterized the early response of human ASCs to GDF6 stimulation, using an unbiased assessment of the temporal transcriptomic changes that occur post‐stimulation. Temporal cellular processes involving differentiation and protein secretion were highlighted, and specific genes validated using qPCR. Mechanisms of lineage commitment were identified, with indication of chondrogenic/NP promotion (SOX9, SOX8, FGFR3, FGF18, SCX, GDF10, FOXF1, and PRG4) and osteogenic (RUNX2) and adipogenic (CEBPA, CEBPD, and NR4A1) inhibition. Perturbation of signaling pathways using inhibitor drugs demonstrated a complex cellular response involving both primary and secondary gene expression, through both Smad1/5/8 and ERK1/2 pathways.

Gene candidates should be further investigated to evaluate their potential in modifying the cellular response to GDF6 or enhancing differentiation, for example through stimulating the FGF18/FGFR3 axis. Identified early differentiation markers would also enable rapid optimization of the minimal GDF6 dose required to drive NP differentiation (potentially lower than the 100 ng/mL used in this study) and rapid screening of patient‐derived ASCs for GDF6‐responsiveness. Such approaches would aid in the translation of a cell‐based therapy for IVD degeneration by ensuring a more patient‐centered personalized medicine approach to treatment and enhancing therapeutic efficacy.

## AUTHOR CONTRIBUTIONS

H.T.J.G provided substantial contribution to data analysis and interpretation and drafted the manuscript; F.E.J.W performed experiments, analyzed, and interpreted the data; L.Z. provided support for analysis of the data; J.A.H and S.M.R secured funding, contributed to the concept and design the study, interpretation of the data, and drafting of the final manuscript. All authors have read and agreed to the published version of the manuscript.

## CONFLICT OF INTEREST STATEMENT

The authors declare that they have no competing interest. J.A.H. is an Editorial Board member of JOR Spine and co‐author of this article. They were excluded from editorial decision‐making related to the acceptance of this article for publication in the journal.

## Supporting information


**DATA S1:** Supporting Information.

## Data Availability

The datasets generated and analyzed during the current study are available from Array Express (https://www.ebi.ac.uk/arrayexpress/; accession number E‐MTAB‐13435).
