# Supplementary material for: Transcriptomic profiling reveals key early response genes during GDF6‐mediated differentiation of human adipose‐derived stem cells to nucleus pulposus cells
Source: JOR Spine. 2024 Jan 19;7(1):e1315. doi: 10.1002/jsp2.1315 (PMC10797253; doi:10.1002/jsp2.1315)
Supplement: Supplementary file 1 — DATA S1: Supporting Information. [file JSP2-7-e1315-s001.pdf]

Supplementary figures & tables

Transcriptomic profiling reveals key early response genes during GDF6-mediated differentiation of human adipose-derived stem cells to nucleus pulposus cells

Hamish T.J. Gilbert[1]*, Francis E.J. Wignall[1]*, Leo Zeef[2], Judith A. Hoyland[1#], Stephen M. Richardson[1#]

[1]Division of Cell Matrix Biology and Regenerative Medicine, School of Biological Sciences, Faculty of Biology, Medicine and Health, University of Manchester, Manchester Academic Health Sciences Centre, Oxford Road, Manchester M13 9PT, UK.

[2]Bioinformatics Core Facility, Faculty of Biology, Medicine & Health, University of Manchester, Oxford Road, Manchester, M13 9PT, UK.

H.T.J.G. hamish.gilbert@manchester.ac.uk; F.E.J.W. frankie_wignall90@hotmail.co.uk; L.Z. leo.zeef@manchester.ac.uk; J.A.H. judith.hoyland@manchester.ac.uk; S.M.R s.richardson@manchester.ac.uk

*Joint first author

#Corresponding authors: J.A.H. judith.hoyland@manchester.ac.uk; S.M.R s.richardson@manchester.ac.uk

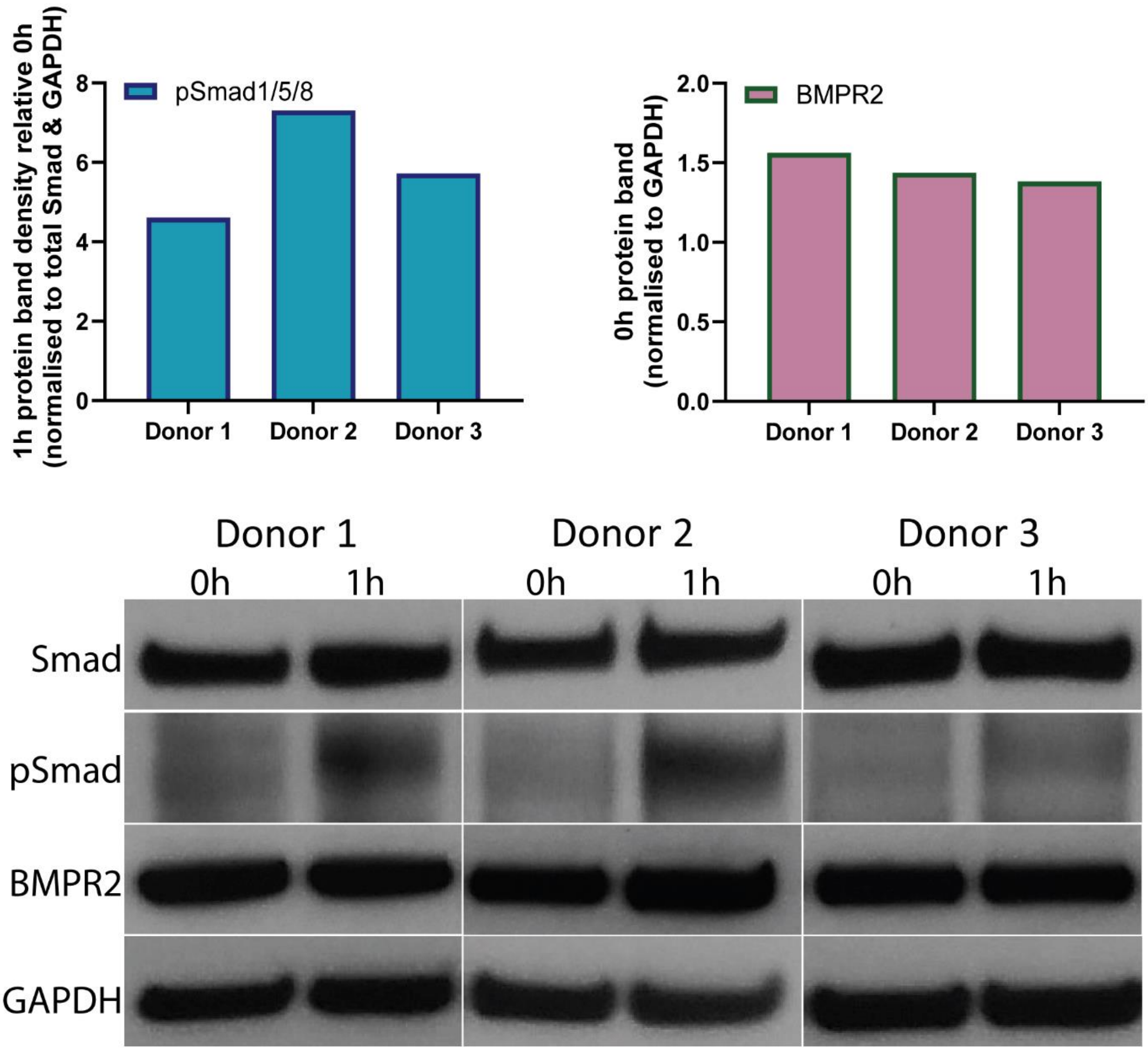

Supplementary Figure 1. Western blot for phosphorylated SMAD (pSMAD) and BMPR2 protein levels. The ratio of pSMAD to total SMAD was calculated and plotted for each donor. Donors 1 – 3 displayed increased phosphorylation of SMAD upon 1 hour of GDF6 stimulation, and so these donors were taken forward for RNA sequencing. BMPR2 levels were calculated normalised to GAPDH at timepoint 0 hours (i.e. no GDF6 stimulation). All three donors had a similar level of expression of BMPR2.

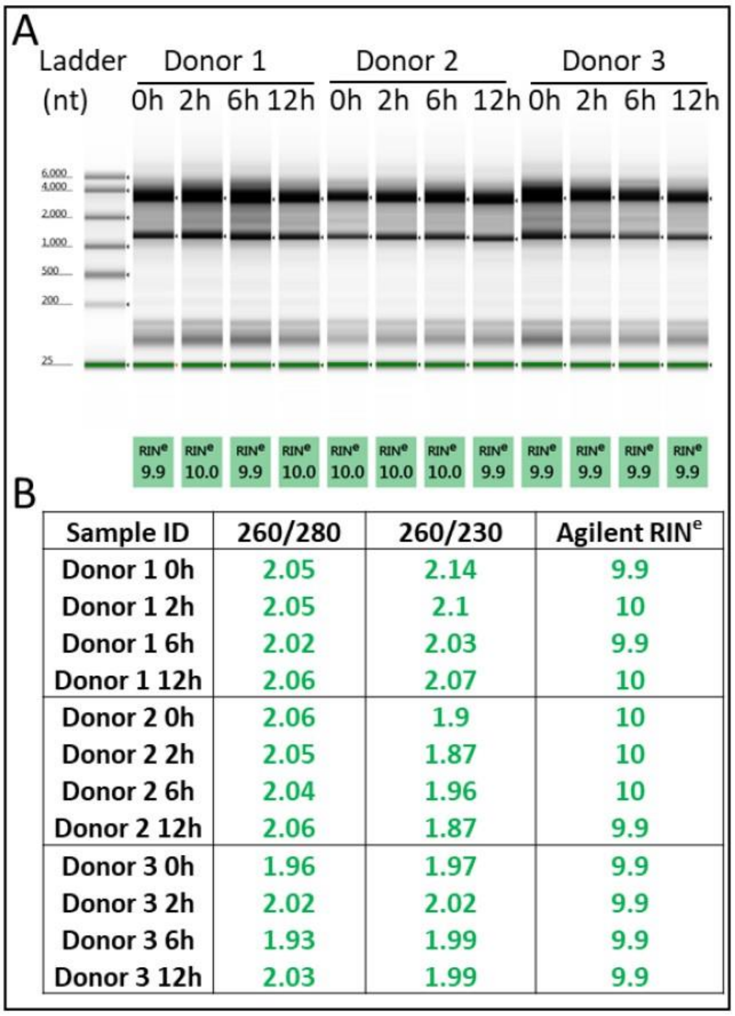

B

Sample ID	260/280	260/230	Agilent RIN^e
Donor 1 0h	2.05	2.14	9.9
Donor 1 2h	2.05	2.1	10
Donor 1 6h	2.02	2.03	9.9
Donor 1 12h	2.06	2.07	10
Donor 2 0h	2.06	1.9	10
Donor 2 2h	2.05	1.87	10
Donor 2 6h	2.04	1.96	10
Donor 2 12h	2.06	1.87	9.9
Donor 3 0h	1.96	1.97	9.9
Donor 3 2h	2.02	2.02	9.9
Donor 3 6h	1.93	1.99	9.9
Donor 3 12h	2.03	1.99	9.9

Supplementary Figure 2. Quality control checks of RNA samples for Cohort 1 donors 1, 2 and 3. Total RNA was quality control checked using **(A)** Agilent TapeStation system for RNA Integrity Number (RIN$_e$ 1-10, 10 being of highest integrity) and **(B)** nanodrop 260/280 and 260/230 ratios for purity assessment.

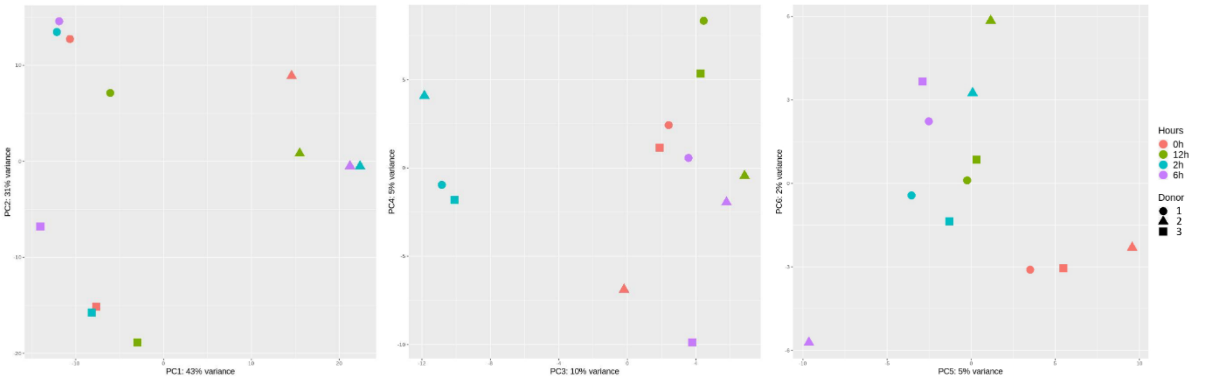

Supplementary Figure 3. Principle component analysis (PCA) showing plots for PC1 to PC6 and samples labelled on donor and timepoint.

Supplementary Table 1: Details of ASC donors used in the study.

Cohort number	Donor number	Age (years)	Sex
1	1	51	Female
1	2	46	Female
1	3	64	Male
2	4	56	Female
2	5	57	Female
2	6	58	Female
2	7	58	Male
2	8	62	Male
2	9	63	Male

Supplementary Table 2: qPCR assay designs.

Gene	TaqMan Assay ID	Accession number	Master mix
**CEBPA**	Hs00269972_s1	NM_001285829.1	TaqMan
**CEBPD**	Hs00270931_s1	NM_005195.3	TaqMan
**EGR1**	Hs00152928_m1	NM_001964.2	TaqMan
**FGF18**	Hs00818572_m1	NM_003862.2	TaqMan
**FGFR3**	Hs00179829_m1	NM_000142.4	TaqMan
**PRG4**	Hs00981633_m1	NM_005807.4	TaqMan
**NR4A1**	Hs00374226_m1	NM_002135.4	TaqMan
**SCX**	Hs03054634_g1	NM_001080514.2	TaqMan
**SOX8**	Hs00232723_m1	NM_014587.4	TaqMan
**SP1**	Hs00916521_m1	NM_138473.2	TaqMan
**Gene**	**Forward, reverse and probe primer sequences**	**Accession number**	**Master mix**
**GDF10**	F: TGCCTAAGATCGTTCGTCCAT<br>R: CCCAAGGGAGTTCATCTTATCG<br>P: TCAGGGCTGTGGGCA	NM_004962.5	TaqMan
**FOXF1**	F: CCGTATCTGCACCAGAAC<br>R: TGGCGTTGAAAGAGAAGA<br>P: CCGAGCTGCAAGGCATCCCG	NM_001451	TaqMan
**PPARG**	F: TCTCCACCTTATTATTCTGA<br>R: CTGTCATAGATAAGCTTCAA<br>P: CGGAAGAAACCCTTGCATCCTT	NM_138712.5	TaqMan
**RUNX2**	F: CGCTGCAACAAGACC<br>R: CGCCATGACAGTAACC<br>P: TGGCCTTCAAGGTGGTAGCCCTC	NM_001024630.4	TaqMan
**SOX9**	F: CAGTACCCGCACTTGCACAAC<br>R: ACTTGTAATCCGGGTGGTCCTT<br>P: AGCTCTGGAGACTTCTGAA	NM_000346.4	TaqMan
**Gene**	**Primer sequences**	**Accession number**	**Master mix**
**EIF2β1**	F: GCCCTCTGCCACCTCAA<br>R: CAACTCCTTCAGCACCAACTA	NM_001414	SYBR Green
**GAPDH**	Sequences proprietary information<br>Anchor nucleotide sequence (1087)	NM_002046	SYBR Green
**MRPL19**	F: CAGGAGATTCAGGTGGTCAAAT<br>R: GCTCTTGTACTACTGGCTTCA	NM_014763	SYBR Green

Supplementary Table 3: Quality control statistics of RNAseq dataset for donor 1 GDF6 stimulation timepoints.

Name	**0h**	**2h**	**6h**	**12h**
Mapping rate	451.59	454.42	465.97	440.56
Number of input reads	28725971	36101529	45173147	28391413
Average input read length	150	150	150	150
Uniquely mapped reads number	27598440	34732413	42916704	27261817
Uniquely mapped reads %	96.07%	96.21%	95.00%	96.02%
Average mapped length	150.14	150.1	150.02	150.11
Number of splices: Total	20765852	25271015	32540042	19391776
Number of splices: Annotated (sjdb)	20705043	25191684	32443730	19330233
Number of splices: GT/AG	20663578	25146546	32384756	19287038
Number of splices: GC/AG	87675	106769	132469	88977
Number of splices: AT/AC	8137	9940	12587	8974
Number of splices: Non-canonical	6462	7760	10230	6787
Mismatch rate per base, %	0.15%	0.16%	0.16%	0.16%
Deletion rate per base	0.00%	0.00%	0.00%	0.00%
Deletion average length	1.51	1.52	1.51	1.53
Insertion rate per base	0.00%	0.00%	0.00%	0.01%
Insertion average length	1.85	1.82	1.83	1.73
Number of reads mapped to multiple loci	980945	1147983	1491113	1057249
% of reads mapped to multiple loci	3.41%	3.18%	3.30%	3.72%
Number of reads mapped to too many loci	4508	6443	6634	5204
% of reads mapped to too many loci	0.02%	0.02%	0.01%	0.02%
% of reads unmapped: too many mismatches	0.00%	0.00%	0.00%	0.00%
% of reads unmapped: too short	0.49%	0.58%	1.67%	0.23%
% of reads unmapped: other	0.01%	0.01%	0.01%	0.01%
Number of reads counted into genes	26,304,002	33,001,463	40,861,035	25,877,230
% of reads counted into genes	92%	91%	90%	91%

Supplementary Table 4: Quality control statistics of RNAseq dataset for donor 2 GDF6 stimulation timepoints.

Name	**0h**	**2h**	**6h**	**12h**
Mapping rate	431.82	449.07	407.5	433.77
Number of input reads	21231352	31185414	27279619	27110445
Average input read length	150	150	150	150
Uniquely mapped reads number	20424803	30109067	26267937	25988552
Uniquely mapped reads %	96.20%	96.55%	96.29%	95.86%
Average mapped length	150.15	150.09	150.16	150.13
Number of splices: Total	14356859	19316323	17803270	18113772
Number of splices: Annotated (sjdb)	14304272	19230899	17733514	18048541
Number of splices: GT/AG	14272612	19199958	17698876	18008257
Number of splices: GC/AG	72346	99416	89093	89632
Number of splices: AT/AC	6879	9995	9316	9501
Number of splices: Non-canonical	5022	6954	5985	6382
Mismatch rate per base, %	0.16%	0.16%	0.16%	0.16%
Deletion rate per base	0.00%	0.00%	0.00%	0.00%
Deletion average length	1.66	1.63	1.63	1.64
Insertion rate per base	0.00%	0.00%	0.00%	0.00%
Insertion average length	1.54	1.54	1.5	1.53
Number of reads mapped to multiple loci	750579	968577	932371	932298
% of reads mapped to multiple loci	3.54%	3.11%	3.42%	3.44%
Number of reads mapped to too many loci	4292	8448	6383	5417
% of reads mapped to too many loci	0.02%	0.03%	0.02%	0.02%
% of reads unmapped: too many mismatches	0.00%	0.00%	0.00%	0.00%
% of reads unmapped: too short	0.23%	0.30%	0.25%	0.67%
% of reads unmapped: other	0.01%	0.02%	0.02%	0.01%
Number of reads counted into genes	19,310,716	27,976,557	24,574,018	24,505,317
% of reads counted into genes	91%	90%	90%	90%

Supplementary Table 5: Quality control statistics of RNAseq dataset for donor 3 GDF6 stimulation timepoints.

Name	**0h**	**2h**	**6h**	**12h**
Mapping rate	442.58	450.75	445.96	440.71
Number of input reads	29259319	33305244	32455837	25463386
Average input read length	150	150	150	150
Uniquely mapped reads number	28228707	32173617	31138497	24547680
Uniquely mapped reads %	96.48%	96.60%	95.94%	96.40%
Average mapped length	150.1	150.13	150.02	150.01
Number of splices: Total	19422581	21399496	22336551	16060170
Number of splices: Annotated (sjdb)	19354456	21318972	22263034	16000897
Number of splices: GT/AG	19316567	21280391	22217297	15968153
Number of splices: GC/AG	89911	101257	101686	78545
Number of splices: AT/AC	9846	10844	10598	8188
Number of splices: Non-canonical	6257	7004	6970	5284
Mismatch rate per base, %	0.16%	0.16%	0.17%	0.17%
Deletion rate per base	0.00%	0.00%	0.00%	0.00%
Deletion average length	1.53	1.55	1.53	1.55
Insertion rate per base	0.00%	0.00%	0.00%	0.00%
Insertion average length	1.57	1.58	1.62	1.53
Number of reads mapped to multiple loci	967747	1059668	1180067	859580
% of reads mapped to multiple loci	3.31%	3.18%	3.64%	3.38%
Number of reads mapped to too many loci	6300	7679	5129	6409
% of reads mapped to too many loci	0.02%	0.02%	0.02%	0.03%
% of reads unmapped: too many mismatches	0.00%	0.00%	0.00%	0.00%
% of reads unmapped: too short	0.18%	0.18%	0.40%	0.18%
% of reads unmapped: other	0.02%	0.02%	0.01%	0.02%
Number of reads counted into genes	26,606,453	30,294,335	29,589,197	22,924,248
% of reads counted into genes	91%	91%	91%	90%
